# Real-World Treatment Patterns, Outcomes, and Healthcare Resource Utilization in Relapsed or Refractory Multiple Myeloma: Evidence from a Medical Record Review in France

**DOI:** 10.1155/2019/4625787

**Published:** 2019-01-29

**Authors:** Huamao Mark Lin, Keith L. Davis, James A. Kaye, Katarina Luptakova, Saurabh P. Nagar, Mohamad Mohty

**Affiliations:** ^1^Millennium Pharmaceuticals, Inc., A Wholly Owned Subsidiary of Takeda Pharmaceutical Company Limited, 40 Landsdowne Street, Cambridge, MA 02139, USA; ^2^RTI Health Solutions, 200 Park Offices Drive, Research Triangle Park, NC 27709, USA; ^3^RTI Health Solutions, 307 Waverley Oaks Road, Suite 101, Waltham, MA 02452, USA; ^4^Department of Haematology, Hôpital Saint-Antoine, Sorbonne University, INSERM UMRs 938, EBMT Paris Study Office, CEREST-TC, Saint-Antoine Hospital, Paris, France

## Abstract

**Background:**

Limited data are available from real-world practices in Europe describing prevailing treatment patterns and outcomes in relapsed/refractory multiple myeloma (RRMM), particularly by cytogenetic risk.

**Methods:**

A retrospective medical record review was conducted in 200 RRMM patients in France. From first relapse, patients were assessed on second-/third-line treatments, progression-free survival (PFS), overall survival (OS), and healthcare utilization.

**Results:**

Fifty-five high risk and 113 standard risk patients were identified. Overall, 192 patients (96%) received second-line therapy after relapse. Lenalidomide-based regimens were most common (>50%) in second line. Hospitalization incidence in high risk patients was approximately twice that of standard risk patients. From Kaplan-Meier estimation, median (95% CI) second-line PFS was 21.4 (17.5, 25.0) months (by high versus standard risk: 10.6 [6.4, 17.0] versus 28.7 [22.1, 37.3] months). Among second-line recipients, 47.4% were deceased at data collection. Median second-line OS was 59.4 (38.8, NE) months (by high versus standard risk: 36.5 [17.4, 50.6] versus 73.6 [66.5, NE] months).

**Conclusions:**

The prognostic importance of cytogenetic risk in RRMM was apparent, whereby high (versus standard) risk patients had decidedly shorter PFS and OS. Frequent hospitalizations indicated potentially high costs associated with RRMM, particularly for high risk patients. These findings may inform economic evaluations of RRMM therapies.

## 1. Introduction

Multiple myeloma (MM) is a malignancy of clonal plasma cells. Worldwide, MM accounts for an estimated 0.8% (114,000) of all new cancer cases annually and 0.9% (63,000) of all cancer deaths annually [1, 2]. In Europe, a recent report suggests there were 38,928 new MM cases and 24,283 MM-attributable deaths in 2012 [3]. Overall, MM accounts for 10% of all hematologic malignancies with median onset age of 68 years [4, 5].

In Europe, autologous stem cell transplant (SCT) is recommended as the standard of care for patients less than 65 years old (although it is often performed in patients over the age of 65 as well) with newly diagnosed MM, which should be preceded by induction therapy aimed at quickly achieving clinical response prior to transplantation [6]. Such induction usually comprises approximately four treatment cycles and available data suggest that three-agent induction regimens, containing at least one novel agent, result in higher response rates than two-agent combinations [7–13]. Patients ineligible for SCT may also be treated with combination chemotherapy containing a novel agent [14–17]. Although MM remains largely incurable, the development of new therapies, including proteasome inhibitors and immunomodulatory drugs, has improved overall survival (OS) to a median of 5 years [18–20]. In the United States, 5-year OS rates have increased from 25% in 1975 to 50% in 2014 [21].

Despite advancements in induction and maintenance therapies leading to improved response rates and OS, virtually all patients with MM eventually relapse and die from disease progression [22]. Following relapse (i.e., relapsed or refractory MM [RRMM]), the mainstays of treatment are immunomodulators (thalidomide, lenalidomide, and pomalidomide), proteasome inhibitors (bortezomib, carfilzomib, and ixazomib), and corticosteroids [23–29]. Other recently approved novel treatments include the monoclonal antibodies daratumumab and elotuzumab, as well as the histone deacetylase inhibitor panobinostat, which have been shown to enhance antineoplastic activity and survival outcomes when used in combination with standard therapies [30–34]. While these novel therapies represent much needed new treatment options, patients with RRMM, once developing refractory disease, still tend to have short responses to treatment and a typical survival expectation of less than one year [23, 35].

To date, little data from routine clinical practice in Europe have been generated to describe prevailing treatment patterns, clinical outcomes, and disease-related healthcare utilization in MM patients after they have relapsed or become refractory to treatment. Furthermore, the extent to which treatment selections, outcomes, and resource use vary according to baseline cytogenetic risk has not been widely explored for RRMM patients in real-world practice settings. Such information may not always comport with what might be expected regarding standards of care, patterns of treatment, and outcomes based on leading academic and clinical research. An assessment of whether, and to what extent, these patterns in real-world settings vary with expectations based on prevailing trial-based guidelines may help inform clinicians and other providers in the ongoing provision of optimal care. This information may also help inform future health technology, economic, and other regulatory assessments of existing and novel RRMM therapies.

## 2. Methods

A retrospective medical record review was conducted in 200 patients with RRMM in France. Patients were selected from the caseloads of 40 hematology/oncology providers practicing across France in a variety of settings: academic, university-affiliated hospitals (35%), nonacademic general hospitals (42.5%), cancer-specialized hospitals (15%), and private community hospitals and clinics (7.5%). For providers with more than 5 patients meeting the study inclusion criteria, selection of 5 patients for the review was based on randomly selected first letters of patients' last names. All patients were aged at least 18 years at initial MM diagnosis and were first diagnosed with RRMM between January 1, 2009, and December 31, 2011. The case identification window terminated in 2011 on the basis of a median survival expectation of 3 to 6 years for RRMM patients receiving active therapy and the need in a retrospective study for an adequate potential follow-up period over which to observe relevant events. All patients were required to have a complete medical record on their treatment history related to MM, beginning with the initial MM diagnosis until death or date of last medical record entry, whichever occurred first. Patients were excluded from the study if they were diagnosed with another concurrent malignancy prior to first diagnosis of RRMM, with the exception of hematologic malignancies secondary to MM (e.g., myelodysplastic syndrome), and adequately treated nonmelanoma skin cancer or in situ neoplasm. All data collection was performed with the formal ethical approval of national competent authorities in France for the conduct of human subjects research.

For purposes of case selection, the participating providers were asked to define RRMM by (1) treatment with a first-line (induction) regimen of chemotherapy with or without SCT and with or without other postinduction/SCT therapies* and* (2) occurrence of disease progression while on or at any time after completion of first-line therapy. The RRMM diagnosis date (i.e., date of first relapse) defined the study index date. Patients were further classified by cytogenetic risk, as follows: (1) high risk: cytogenetic abnormalities del(17p), t(4:14), or t(14;16); (2) standard risk: all patients with known cytogenetics not classified as high risk; and (3) unknown risk: patients with unknown cytogenetics. To obtain an adequate sample of high risk patients for selected stratified analyses, a soft quota was imposed on patient selection to ensure that between 20% and 30% of the final study sample had high risk disease.

From date of first relapse, patients were retrospectively assessed for second- and third-line treatment regimens received, treatment duration, reasons for discontinuation, and MM-related healthcare utilization, as well as OS and progression-free survival (PFS) from initiation of second-line treatment. PFS was calculated as time from second-line treatment initiation until the earliest of clinical progression during treatment, switch to new therapy line, or death (during or after treatment) if no additional treatment lines were initiated. All analyses were descriptive and exploratory in nature. OS and PFS were descriptively analyzed using the Kaplan-Meier method.

## 3. Results

**Figure 1:**
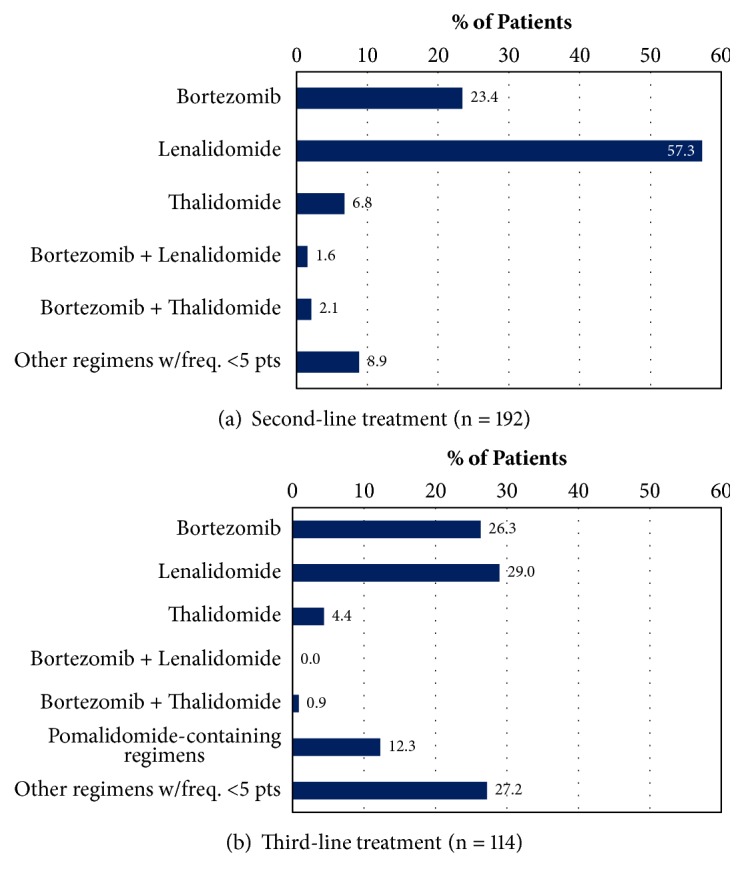
Second- and third-line treatment regimens. Note: regimen compositions listed are irrespective of concomitant dexamethasone use.

As shown in Table 1, a total of 55 high risk and 113 standard risk patients were identified; risk category was unknown or unassessed for 32 patients. Mean (SD) age at first relapse was 66.3 (8.9) years and 61.5% of patients were male. Eighty-one patients (41%) received SCT (autologous SCT [n = 68] or tandem autologous SCT [n = 13]) as part of their overall first-line treatment course. Bortezomib-based systemic treatments were the most common first-line induction therapy, being observed in nearly two-thirds of all patients. In total, as shown in Figure 1, 192 patients (96%) received additional systemic therapy (i.e., second-line treatment) after first relapse. Lenalidomide-based regimens (+/- dexamethasone) were most common (>50% of patients) in second-line treatment, regardless of baseline cytogenetic risk; the median duration of these regimens was approximately 1 year over a median of 12.5 cycles. Bortezomib-based regimens (+/- dexamethasone) were next most common in second-line treatment, with a median duration of approximately 6 months over a median of 6 cycles. Among the 192 patients initiating second-line therapy, 114 (59%) also received third-line treatment; regimen compositions were more varied in the third line.

**Figure 2:**
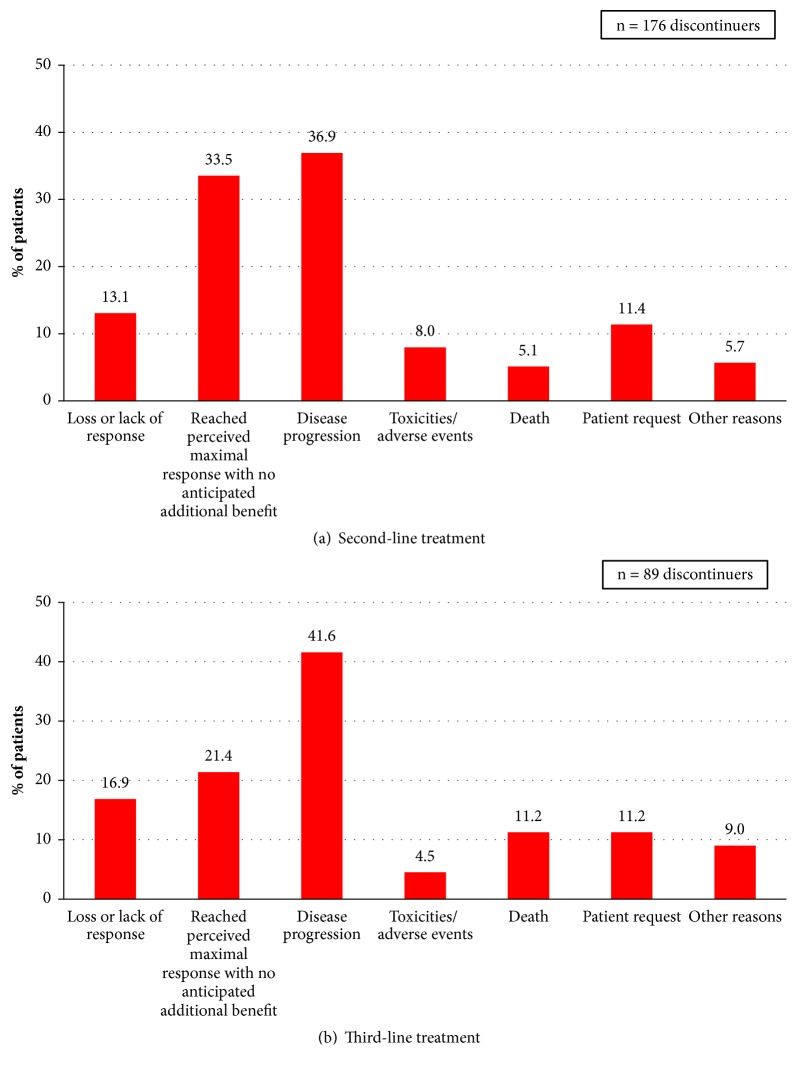
Reasons for treatment discontinuation.

Table 2 depicts observed second- to third-line treatment sequencing in the patients reviewed here. Among patients initiating a bortezomib-based regimen in second-line, lenalidomide (+/- dexamethasone) was the most common third-line treatment, followed by best supportive care (i.e., active, antineoplastic third-line treatment not initiated). Among second-line lenalidomide recipients, nearly half did not initiate a third-line treatment, having received best supportive care only. Relatively few patients switched to bortezomib in third-line treatment following discontinuation of second-line lenalidomide. Most patients (n = 176, 92%) had discontinued second-line treatment by the time the medical record review was conducted, most often due to disease progression (37%), to reaching a perceived maximal response with no additional benefit expected (33%), and to lack or loss of response (13%) (‎Figure 2(a)). Among patients discontinuing third-line treatment (n = 89, 78%), the leading reason for discontinuation was disease progression (42%), followed by attainment of a perceived maximal response with no additional benefit expected (21%) (Figure 2(b)).

As shown in Table 3, hospitalization incidence in patients with high risk MM (0.34 per person-year) was approximately twice that of patients with standard risk (0.15 per person-year). The use of outpatient services was roughly equal between standard- and high risk patients. By line of therapy, as indicated in Table 4, healthcare utilization incidence was generally higher in third-line therapy as compared with second-line. The most frequently utilized health service category was hospital outpatient visits, particularly for patients treated with bortezomib-containing regimens.

**Figure 3:**
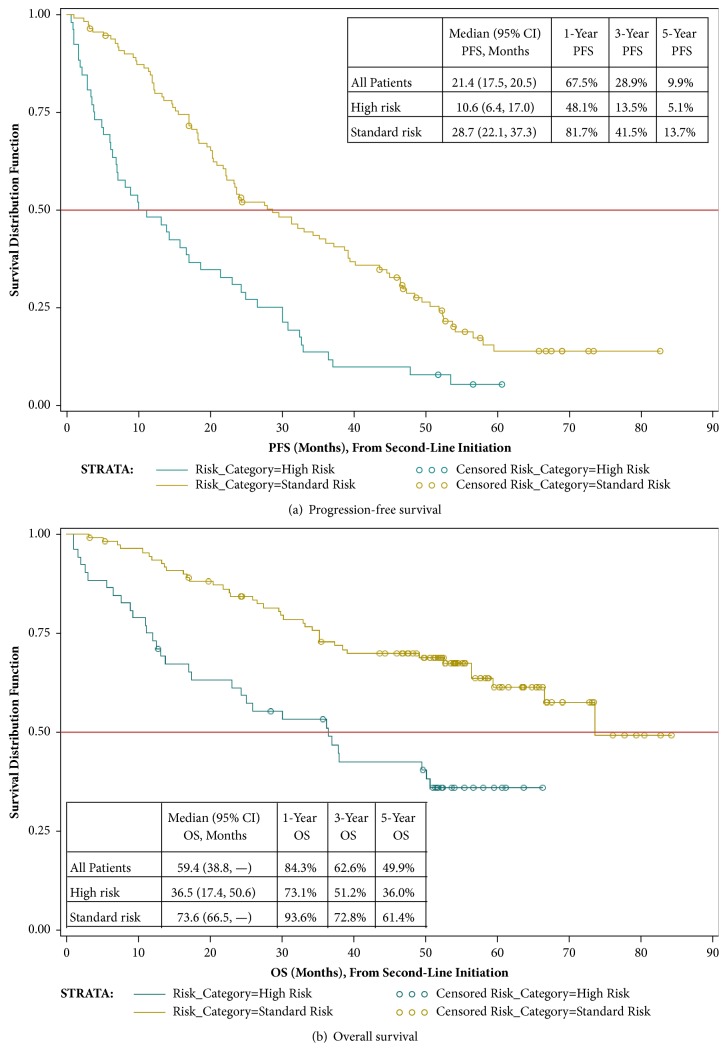
Progression-free and overall survival from initiation of second-line treatment.

**Figure 4:**
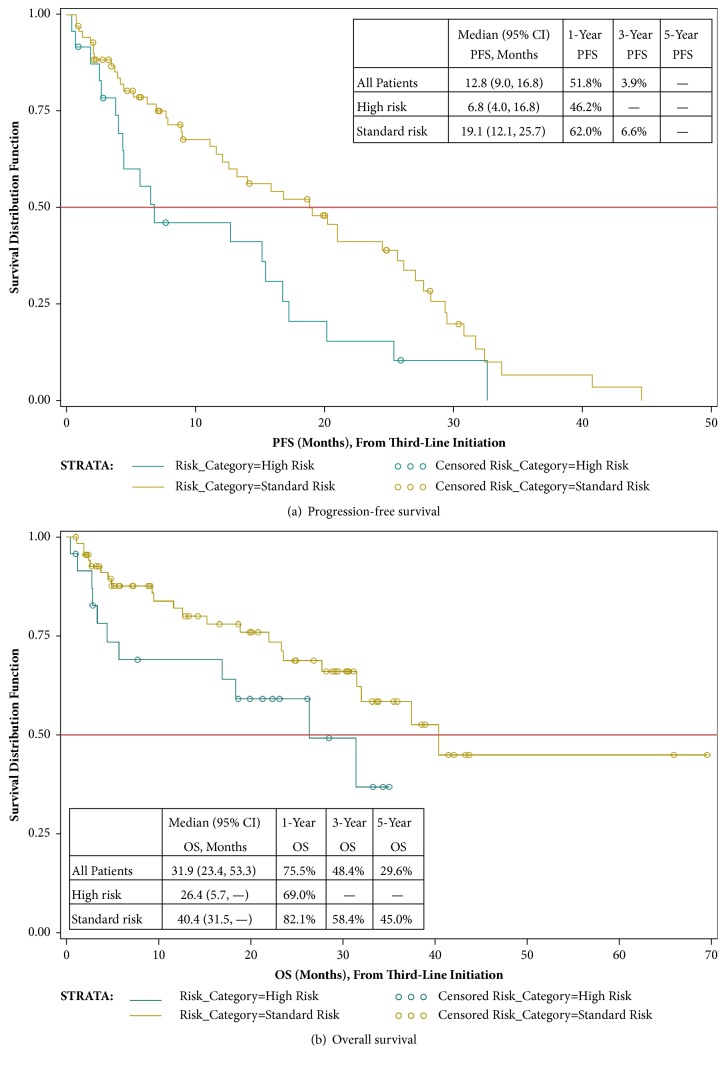
Progression-free and overall survival from initiation of third-line treatment.

Clinical progression during second-line treatment occurred for 47.4% of the 192 patients initiating second-line treatment; 84.9% of patients had experienced a progression event by the time of their last available medical record over a median total follow-up duration of 52 months. Based on Kaplan-Meier estimation, the median (95% confidence interval [CI]) PFS from second-line treatment initiation was 21.4 (17.5, 25.0) months for all patients combined (Figure 3(a)); median PFS was substantially lower for high risk patients (10.6 [6.4, 17.0] months) and unknown risk patients (9.8 [4.0, 25.2] months) as compared with standard risk patients (28.7 [22.1, 37.3] months). Among all patients initiating a second-line treatment (n = 192), 47.4% were deceased at the time of data collection. The median (95% CI) OS from second-line treatment initiation was 59.4 (38.8, NE) months (Figure 3(b)). Median (95% CI) OS from second-line initiation in high risk and unknown risk patients (36.5 [17.4, 50.6] and 32.5 [11.9, 38.8] months, respectively) was substantially lower than in standard risk patients (73.6 [66.5, NE] months). PFS and OS for third-line treatment recipients are presented in Figures 4(a) and 4(b).

## 4. Discussion

Treatment selections in the relapse setting for patients with MM depends on several parameters such as age, performance status, comorbidities, the number of prior treatment lines, available remaining treatment options, and interval since completion of last therapy. The European Medicines Association (EMA) has approved lenalidomide in combination with dexamethasone, as well as bortezomib either alone or in combination with pegylated doxorubicin, as recommended therapy for RRMM based on results of numerous trials [36–39]. Current RRMM treatment guidelines from the European Society for Medical Oncology (ESMO) acknowledge that in routine practice bortezomib is the most frequently used agent, typically in combination with dexamethasone [40]. Among the French population reviewed here, however, the EMA-recommended second-line combination of lenalidomide/dexamethasone was, in fact, the most commonly reported regimen in second-line (53.7%), indicating that treatment selections in routine practice were consistent with guideline-based expectations.

Second-line treatment duration was generally less than 1 year, indicating an unmet need in relapsed MM patients in light of the primary reasons cited for discontinuation (disease progression or loss of response, no perceived additional benefit, and toxicities). Second- and third-line treatment durations were longer for oral lenalidomide-based regimens (median: 12.5 and 10 cycles for second- and third-line, respectively) as compared with intravenous bortezomib regimens (median: 6 cycles in both second- and third-line). Additional research is needed to further explore whether oral treatments in the relapse setting confer a potential treatment persistence benefit due to the simpler administration route.

In our study sample, nearly all patients (192 of 200 [96%]) received at least one line of additional chemotherapy (i.e., second-line therapy) after first relapse. Of these patients, only 114 (59.4%) went on to receive third-line therapy. This proportion is somewhat lower than expected. Among the 86 second-line initiators who did not receive third-line treatment, 53 (61.6%) died before a third-line could be initiated, including 23 patients who died while on second-line therapy and 30 patients who died after completion of second-line and before a next line could be started. Of the remaining patients who did not initiate a third-line therapy, 16 were still on their second-line treatment at last follow-up and 17 were censored (having not yet initiated a third-line treatment at last available follow-up). Thus, death was the predominant reason for the observed lack of third-line treatment, but censoring (loss to follow-up) was also a substantial factor.

MM-related hospitalizations and emergency department visits were common after first relapse, indicating a potentially high cost burden borne by patients with RRMM. Hospitalization incidence was nearly 50% higher for patients with high risk cytogenetics as compared with those with standard risk. The introduction of additional, more efficacious treatment options for RRMM that reduce the incidence of hospitalizations related to disease progression or complications may therefore present cost savings to health systems. Additional research in the area of MM-related costs and cost-effectiveness is needed to formally assess the economic impact of newer therapies.

Recent trial-based estimates of PFS and OS after MM relapse are wide-ranging and dependent upon specific patient characteristics, including treatment regimen administered, number of previous regimens received, and baseline risk category, among others factors. A recent review article by Nooka et al. [41] highlights data from numerous interventional trials of various treatment regimens used in second- and later-line treatment of MM. From this review, median PFS from second-line treatment initiation in patients with no prior treatment other than induction therapy (+/- SCT) ranged from 8 months for bortezomib monotherapy and bortezomib/siltuximab combination therapy [42] to 18 months for bortezomib/thalidomide/dexamethasone triplet therapy [43]. For patient populations that were more heavily pretreated (i.e., two or more prior therapy lines, with PFS estimated from third-, fourth-, or later-line treatment initiation), Nooka et al. reports PFS estimates that were predictably shorter but still wide ranging; this includes one estimate of a 27-month PFS for elotuzumab/lenalidomide/dexamethasone [44]. The ASPIRE study reported a 26-month PFS for carfilzomib/lenalidomide/dexamethasone in patients with 1 to 3 previous lines of therapy (median: 2) before relapse [45]. Compared with the PFS estimates cited by Nooka et al., our findings (median PFS: 21 months from second-line initiation) were more consistent with those of Garderet et al. [43] who reported a median PFS of 18 months for patients who, as in our study, had received only one prior line of therapy. We estimated a median third-line PFS (in patients with ≥2 prior lines of therapy) of 12.8 months (Figure 4), which fell within a range of several third-line PFS estimates summarized by Nooka et al. for patients with 2 prior therapy lines: median 9.5 months for lenalidomide/bortezomib/dexamethasone [46], 11.6 months for pomalidomide/dexamethasone [28], and 16.1 months for cyclophosphamide/prednisone/lenalidomide [47].

OS in both MM generally and in RRMM specifically has seen a marked improvement from the year 2000 onward [48]. One study, for example, explored the use of lenalidomide-based regimens in RRMM, finding an encouraging median OS of 37 months for lenalidomide/bortezomib with or without dexamethasone [49]. Other studies have also reported a median OS after relapse exceeding 30 months even in patients with multiple prior therapy lines [36, 38, 42, 46, 50]. In our study, median (95% CI) OS from second-line initiation varied by risk category: 36.5 (17.4, 50.6) months in high risk patients, 32.5 (11.9, 38.8) months in unknown risk patients, and 73.6 (66.5, NE) months in standard risk patients. Median OS from relapse (i.e., at or close to second-line treatment initiation) is frequently not reached in trials of RRMM treatments, even in patient populations with multiple prior therapy lines (and thus, relapses) who should be predisposed to shorter survival and higher likelihood of a death event. In Nooka et al.'s review, for example, median OS was not reached in 16 of 39 studies reviewed, the majority of which involved patients who had received multiple prior lines of therapy. OS from second-line treatment initiation in high risk and unknown risk patients in our study appears consistent with the literature cited above. However, our OS estimate for standard risk patients is above the range reported from these studies, due potentially to the small sample size for the review conducted here as well as differing selection criteria between real-world studies such as this one and interventional clinical trials.

Our analysis was subject to several limitations. First, as is inherent to retrospective medical record reviews, assessments of disease progression were not protocol driven, particularly with regard to the timing of evaluations of disease progression. It is possible that the participating physicians, some of whom likely do not frequently participate as investigators in clinical trials, assessed clinical progression at less frequent intervals than would otherwise be required in a clinical trial, and progression events may have been identified somewhat later than they would have otherwise. In this case, PFS estimates may have been upwardly biased. For this reason, findings regarding the endpoints of PFS may not be directly comparable to those observed in clinical trials. Second, while measures were taken to randomize patient selection for the medical record review, the physicians and patients selected for study inclusion still represent a practical nonrandomized sample. Our study findings therefore may not be generalizable to the overall RRMM population and the noted comparisons to previous clinical trials should be made with caution based on the differing and generally more stringent inclusion criteria used in the interventional studies reviewed. Our study is also be limited by its relatively small sample size, which may further reduce generalizability of the results. Third, the criteria used for cytogenetic risk classification were based on consensus definitions contemporaneous to the study entry window (2009-2011) for the cohort studied here [51]. Risk classifications have since evolved, but due to the retrospective nature of our study, it was not possible to impose alternate definitions based on alternate, more currently utilized criteria such as those also incorporating ISS staging. Fourth, our study excluded patients with a concurrent malignancy (other than nonmelanoma skin cancer and hematologic malignancy secondary to MM) prior to first diagnosis of RRMM. As noted in several studies, the incidence of second primary malignancies (SPMs) in MM patients receiving lenalidomide induction or maintenance therapy is high (~17%, as reported in one recent large randomized trial [52]). This exclusion was imposed in our study so that treatments specifically directed toward relapsed MM (rather than an SPM) could be more clearly discerned, as the data were historical in nature and treatments were not assigned according to a predefined protocol. Although there was no limitation on later development of an SPM, it is possible that this exclusion could have introduced some upward bias to our estimates of OS and PFS. Finally, this study was not designed to assess comparative effectiveness of alternative RRMM treatments, as the observed treatment regimens occurred in the course of usual care and were not randomized. We therefore cannot present direct comparisons of clinical outcomes (PFS and OS) between alternative treatment regimens initiated as second-line therapy.

## 5. Conclusions

To date, only limited data from real-world clinical settings in Europe have been generated describing current practice patterns, outcomes, and healthcare utilization in MM patients in the relapse/refractory setting. Our study found that treatment practices in real-world settings to manage relapsed MM, starting with second-line treatment, generally align with ESMO guidelines in that lenalidomide/dexamethasone was the most commonly reported second-line regimen (53.7% of patients). Furthermore, the importance of initial MM risk classification as a prognostic factor in RRMM was apparent in this retrospective review, whereby (as in previous studies) patients with high risk disease had decidedly less favorable PFS and OS than patients with standard risk. Finally, based on frequent hospitalization and emergency department visits in the relapse setting, our study demonstrates the potentially high cost burden associated with RRMM and the additional cost burden that may be incurred by patients with high risk MM as compared with standard risk. Taken together, findings from this study address important literature gaps in RRMM and may help inform future economic (e.g., cost and cost-effectiveness) evaluations of novel RRMM therapies.

## Figures and Tables

**Table 1:** Baseline demographics and clinical characteristics.

	**All Patients**		**Risk Category at Initial MM Diagnosis** ^**a**^					
			**High Risk**		**Standard Risk**		**Unknown Risk**	
	**n**	%	**n**	%	**n**	%	**n**	%
**All patients, n (**%**)**	200	100.0	55	100.0	113	100.0	32	100.0

**Age (years) at initial MM diagnosis**								

Mean (SD)	64.5	9.2	62.9	10.6	64.5	8.3	67.1	9.5

< 65 years, n (%)	103	51.5	33	60.0	54	47.8	16	50.0

≥ 65 years, n (%)	97	48.5	22	40.0	59	52.2	16	50.0

**Age (years) at RRMM diagnosis**								

Mean (SD)	66.3	8.9	64.4	10.5	66.6	7.9	68.7	9.1

< 65 years, n (%)	85	42.5	31	56.4	45	39.8	9	28.1

≥ 65 years, n (%)	115	57.5	24	43.6	68	60.2	23	71.9

**Sex, n (**%**)**								

Male	123	61.5	33	60.0	74	65.5	16	50.0

Female	77	38.5	22	40.0	39	34.5	16	50.0

**ISS stage at initial MM diagnosis, n (**%**)**^**b**^								

Stage I	26	13.0	4	7.3	14	12.4	8	25.0

Stage II	81	40.5	19	34.6	53	46.9	9	28.1

Stage III	88	44.0	31	56.4	42	37.2	15	46.9

Unknown	5	2.5	1	1.8	4	3.5	—	—

Impaired renal function, n (%)	12	6.0	6	10.9	4	3.5	2	6.3

**Received stem cell transplant as part of first-line (induction) therapy, n (**%**)**								

Autologous SCT	68	34.0	19	34.6	40	35.4	9	28.1

Tandem (double) autologous SCT	13	6.5	1	1.8	8	7.1	4	12.5

SCT not received	119	59.5	35	63.6	65	57.5	19	59.4

**First-line (induction) systemic treatment regimens, n (**%**)**								

Bortezomib + dexamethasone	55	27.5	7	12.7	37	32.7	11	34.4

Bortezomib + thalidomide + dexamethasone	27	13.5	13	23.6	11	9.7	3	9.4

Melphalan + prednisone + bortezomib	26	13.0	8	14.6	14	12.4	4	12.5

Melphalan + prednisone + thalidomide	24	12.0	6	10.9	12	10.6	6	18.8

Vincristine + doxorubicin + dexamethasone	18	9.0	1	1.8	14	12.4	3	9.4

Melphalan + prednisone	14	7.0	5	9.1	9	8.0	—	—

Bortezomib + cyclophosphamide + dexamethasone	12	6.0	8	14.6	3	2.7	1	3.1

Other induction regimens with frequency of <5 patients	24	12.0	7	12.7	13	11.5	4	12.5

**Vital status at chart abstraction date, n (**%**)**								

Alive	101	50.5	20	36.4	72	63.7	9	28.1

Deceased	99	49.5	35	63.6	41	36.3	23	71.9

**Duration (months) of follow-up, from RRMM diagnosis to death/last available medical record, median**	52		38		53		32	

SCT = stem cell transplant, SD = standard deviation, and ISS = International Staging System.

^a^High risk: gene rearrangements del(17p), t(4;14), or t(14;16). Standard risk: all patients with known cytogenetics not classified as high risk. Unknown risk: patients with unknown cytogenetics.

^b^Stage I: serum *β*2-microglobulin < 3.5 mg/L and serum albumin ≥ 3.5 g/dL. Stage II: not stage I or III. Stage III: serum *β*2-microglobulin ≥ 5.5 mg/L.

**Table 2:** Second- to third-line treatment sequencing.

**Second-Line Regimens** **∗**			**Third-Line Regimen Compositions** ^**a**^						
**Regimen Composition**	**No. Patients**		**BOR**	**LEN**	**THAL**	**BOR + LEN**	**BOR + THAL**	**Other Regimens**	**No Treatment**
Bortezomib (BOR)	45	→	1	22	2	0	0	6	14

Lenalidomide (LEN)	110	→	24	3	1	0	1	33	48

Thalidomide (THAL)	13	→	2	1	0	0	0	3	7

BOR + LEN	3	→	1	2	0	0	0	0	0

BOR + THAL	4	→	0	3	0	0	0	0	1

Other regimens	17	→	2	2	2	0	0	3	8

Total	192	→	30	33	5	0	1	45	78

^a^Listed regimen compositions are irrespective of concomitant dexamethasone use.

**Table 3:** Health care utilization from first relapse to last available follow-up.

	**All Patients**		**Risk Category at Initial MM Diagnosis** ^**a**^			
			**High Risk**		**Standard Risk**	
	**n**	%	**n**	%	**n**	%
**All patients, n (**%**)**	200	100.0	55	100.0	113	100.0

**Inpatient hospitalizations**						

Had ≥ 1 hospitalization, n (%)	70	35.0	23	41.8	32	28.3

No. hospitalizations per person-year	0.24		0.34		0.15	

**Emergency department visits**						

Had ≥ 1 visit, n (%)	54	27.0	16	29.1	28	24.8

No. visits per person-year	0.23		0.25		0.15	

**Office visits**						

Had ≥ 1 visit, n (%)	62	31.0	16	29.1	33	29.2

No. visits per person-year	1.20		1.07		0.97	

**Hospital outpatient/day visits**						

Had ≥ 1 visit, n (%)	136	68.0	38	69.1	77	68.1

No. visits per person-year	4.65		4.68		4.60	

^a^High risk: gene rearrangements del(17p), t(4;14), or t(14;16). Standard risk: all patients with known cytogenetics not classified as high risk.

**Table 4:** Health care utilization during active treatment.

	**Second-Line**				**Third-Line**			
	**Bortezomib-Containing Regimens**		**Lenalidomide-Containing Regimens**		**Bortezomib-Containing Regimens**		**Lenalidomide-Containing Regimens**	
	**n**	%	**n**	%	**n**	%	**n**	%
**All patients, n (**%**)**	45	100.0	110	100.0	30	100.0	33	100.0

**Inpatient hospitalizations**								

Had ≥ 1 hospitalization, n (%)	9	20.0	25	22.7	8	26.7	5	15.2

No. hospitalizations per person-year	0.19		0.15		0.43		0.20	

**Emergency department visits**								

Had ≥ 1 visit, n (%)	8	17.8	17	15.5	6	20.0	7	21.2

No. visits per person-year	0.19		0.15		0.32		0.47	

**Office visits**								

Had ≥ 1 visit, n (%)	8	17.8	35	31.8	8	26.7	5	15.2

No. visits per person-year	0.62		1.59		2.17		1.42	

**Hospital outpatient/day visits**								

Had ≥ 1 visit, n (%)	34	75.6	66	60.0	22	73.3	22	66.7

No. visits per person-year	6.03		3.99		12.16		6.38	

## Data Availability

In compliance with the noted ethics requirements and approvals in France regarding data privacy in human subjects research, the patient-level data collected for this study are not publicly available.
